# Evaluation of Normal Renal Size and its Influencing Factors: A Cross-Sectional Study on the Adult Population of Mashhad

**DOI:** 10.22088/cjim.13.3.623

**Published:** 2022

**Authors:** Masoud Pezeshki Rad, Bita Abbasi, Niloufar Valizadeh, Farbod hatami, Fariba tohidinezhad, Zahra Gharehbaghi

**Affiliations:** 1 Department of Radiology, Mashhad University of Medical Sciences, Mashhad, Iran; 2 Cardiovascular Diseases Research Center, Birjand University of Medical Sciences, Birjand, Iran; 3 Department of Medical Informatics, Mashhad University of Medical Sciences, Mashhad, Iran

**Keywords:** Kidney size, Ultrasound, Anthropometric characteristics, Renal function

## Abstract

**Background:**

The normal range of kidney size is a controversial issue among different populations given to its impressibility by multiple factors, therefore, this study aimed to provide valid reference ranges for kidney dimensions in the adult population of Mashhad. Also, we assessed the association of kidney size characteristics with some personal predisposing factors.

**Methods:**

This cross-sectional study was conducted on 938 healthy individuals. Ultrasound measurement, physical examination, and laboratory analysis were performed. Demographic, dietary, and anthropometric data were obtained. The variables were categorized into 5 groups each, and data analysis were performed using the following statistical tests: Pearson correlation test, variance analysis, t-test, and chi-square test. A value of p<0.05 was considered statistically significant.

**Results:**

Weight had the most association with kidney size followed to a lesser extent by height and age. Even after adjustment for other confounding variables, weight remained as an independent factor, while this effect was resolved for height and age. Also, all values for renal function, body bio-impedance, blood pressure components, and water intake were notably correlated with kidney size.

**Conclusion:**

This study determined the normal kidney size in healthy adults. We also declared the normal range of kidney size is a dynamic concept and should be assessed for each individual separately according to their personal determinative factors.

Renal size is an important and useful parameter for diagnosis, decision making, and clinical management of renal disorders regarding its close relationship to kidney function (1). The optimal ranges for the healthy adults’ kidney size have been largely expressed by previous reports; herein, approximate estimations of each of the following parameters are 2.5-3 centimeters (cm) for thickness, 90-130 cm for length, and 5-5.7cm for width (2,3). Studies have shown remarkable differences in estimated normal kidney size between populations, which has been attributed to ethnic and racial diversity (4). Timely diagnosis and treatment of chronic kidney disease which is mainly known by a decrease in renal size can prevent the disease aggravation and its adverse consequents (5). Moreover, regarding anatomic landmarks, kidney size parameters are precisely required when performing a renal biopsy under ultrasound guidance. In the past several decades, ultrasonography has played an important role in evaluating renal size; as it is an inexpensive, accurate, non-invasive, radiation-free and readily available modality to determine length, width, and thickness of the organ (6).

Although, ultrasound is a well-established diagnostic method, yet there are insufficient data on the aspects of normal kidney size and its influencing factors in the healthy adult population. To the best of our knowledge, this is the first study in our region that aimed to evaluate the issue on a large sample volume.

## Methods

After approval by the local Ethics Committee from the Mashhad University of Medical Sciences Review Board (IR.MUMS.MEDICAL.RE.1397.104), this cross-sectional study was conducted at the radiology department of Imam Reza hospital in Mashhad during 2018-2019. Samples were selected by the available-sampling method and informed consent was obtained from all participants. Inclusion criteria were: age more than 18 years, serum creatinine ≤1.5 milligrams/deciliter (mg/dL), fasting blood sugar <110 mg/dL, and normal kidney appearance at the ultrasound.

The exclusion criteria included chronic or acute kidney disease, situations affecting kidney condition such as diabetes, hypertension, pregnancy, abnormalities in kidney anatomy such as renal cyst, and the patients who did not complete the study for any reason.

A total of 1000 individuals volunteered for the current study while 62 patients were excluded, the staff of Mashhad University of Medical Sciences who voluntarily participated were examined. The administrative unit made contact with the cases and the individuals selected their desired time and date and after registering the appointment, they were referred to the research unit. Each person received an identification code before the investigation.

**Figure 1:**
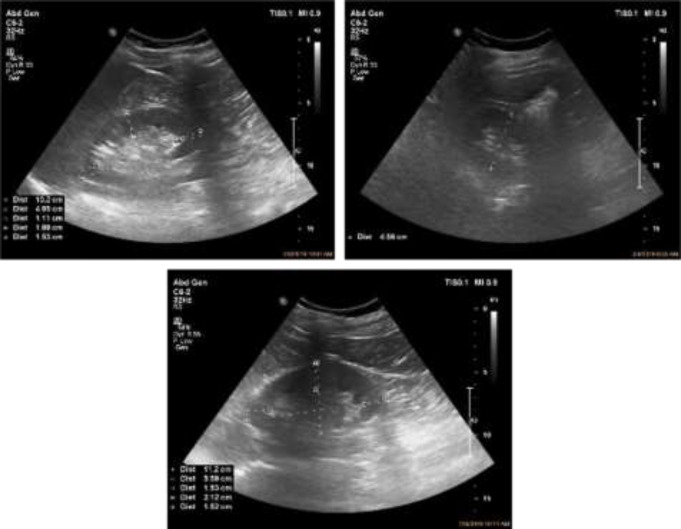
Sonographic measurement of the kidney length and width and AP diameter

Thirteen stations were defined for data collection. For all the participants, body composition analysis was performed via the bioimpedance method, by Bioelectrical Impedance Analysis Equipment, which evaluates the amount of body protein, intracellular and extracellular water, and minerals based on the absorption of electromagnetic rays. Likewise, serum creatinine and urea levels were evaluated, and also anthropometric characteristics including height, weight, body mass index (BMI), body surface area (BSA), and abdominal circumference were recorded. Each individual underwent renal ultrasonography to measure kidney length, width, thickness, and anteroposterior diameter. The procedure was performed using the Philips Affinity 50G device with a C6-2 Convex probe and a frequency of 3.2 HZ in longitudinal and transverse view point in the supine position. Parenchymal thickness was measured in three (upper, middle, and lower) poles for each individual (figure 1).

The results were collected in a pre-designed checklist. Data analysis was performed via SPSS software Version 21 (SPSS Inc., Chicago, IL, USA) using statistical methods including descriptive and inferential statistics. Data were presented as the mean ± standard deviation and the t-test, chi-square, and variance tests were used to identify relationships. Bivariate and multivariate analysis was performed to examine the effect of possible confounders. A value of P<0.05 was considered statistically significant. The datasets supporting the conclusions of this article are available in the radiology department of Imam Reza Hospital data base (Mashhad, Iran) repository. Furthermore, the datasets analyzed during the current study are available from the corresponding author on reasonable request.

## Results

For the present study, we analyzed the data collected from 938 individuals, of which 444 were males and 494 were females. The average age of men was 46 ± 8.1 years and for women was 43.4±6.9. Our cases were mostly Persian, while no significant difference was noted among different ethnicities including Persian, Turk, Kurd, and others regarding gender. While all the anthropometric, bio-impedance, biochemistry, blood pressure, and ultrasound measures mentioned were higher in the male group (p<0.001), BMI and dietary intakes did not have a notable difference between two gender groups (table 1).

Findings from ultrasonic measurements revealed significantly greater values for Right Renal Length (RRL), Left Renal Length (LRL), Right Renal Width (RRW), and Left Renal Width (LRW) in men compared to women (p<0.001). A significant difference in renal size was noted in female gender when considering age groups, so, the age group of 41-50 years had an increase in both right (104.7±8.4 mm, p=0.004) and left (104.9±10.8 mm, P=0.039) kidney length in comparison to other age groups (table 2).

**Figure 2:**
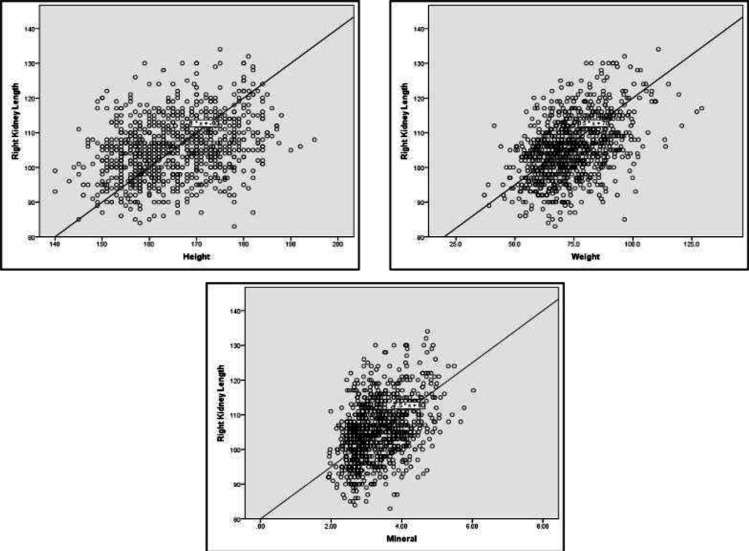
The Linear relationship of the height, weight, and minerals with right kidney sizes (length, width, and AP diameter)

**Figure 3:**
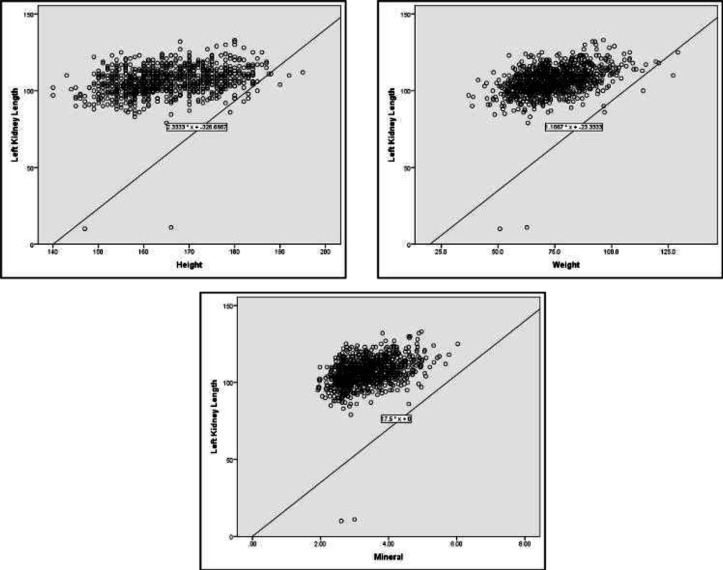
The linear relationship of the height, weight, and minerals with left kidney sizes (length, width, and AP diameter)

In both kidneys, the mean values for parenchymal thickness and renal length were statistically higher in men and women over 180 cm and 170 cm of height, respectively (table 3). Our results demonstrated that in men weighing over 100 kg, an increase in all parameters of kidney size was observable (p<0.05). In the women group, tests showed a significant association between the weight and all kidney size parameters except for Left Renal Parenchymal Thickness (LRP) (p<0.05). The mean width and parenchymal thickness of both kidneys were notably higher than other weight groups in women weighing 90-100 kg and over 100 kg respectively (table 4). We performed the Pearson correlation test to assess the role of possible factors contributing to the variation of the kidney sizes. Results of the Pearson test confirmed a significant positive correlation between all the variables of the current study including age, anthropometric characteristics, body composition components, blood pressure values, laboratory values, amount of water intake, and kidney size parameters. This effect was not seen in the case of salt intake. Details are presented in table 5. Based on the findings of the single-variable regression test (table 6), the relationship between height, weight, abdominal circumference, body water, minerals, protein, systolic, and diastolic blood pressure and Mean Arterial Pressure (MAP) with kidney length was significant. Also, based on the findings of multiple regressions analysis, the relationships of 3 variables (weight, height, and minerals) with kidney length were significant (figures 2, 3). The value of the determination coefficient for the final model was 0.27 (β = 97.1), which indicates the prediction of 27% of changes in kidney thickness by these variables.

**Table 1:** Basic demographic and clinical characteristics of the population (N=938)

Variable	**Male **(N=444)	Female (N=494)	P-value
**Age (year)**	46.0 ± 8.1	43.4 ± 6.9	<0.001
**Anthropometric**			
**Height (cm)**	172.9 ± 6.9	158.3 ± 5.9	<0.001
**Weight (kg)**	80.6 ± 12.5	68.3 ± 11.3	<0.001
**BMI (kg/m** ^2^ **)**	26.9 ± 3.6	27.3 ± 4.3	0.185
**BSA (m** ^2^ **)**	1.9 ± 0.2	1.7 ± 0.1	<0.001
**Abdomen Circumference(cm)**	96.0 ± 10.6	91.8 ± 10.2	<0.001
**Bio-impedance**			
**Body Water (L)**			
**Total**	42.4 ± 5.6	29.8 ± 3.6	<0.001
**ICW**	26.4 ± 3.5	18.4 ± 2.2	<0.001
**ECW**	16.0 ± 2.1	11.5 ± 1.4	<0.001
**Mineral (kg)**	3.9 ± 0.6	2.8 ± 0.3	<0.001
**Protein (kg)**	11.4 ± 1.5	7.9 ± 1.0	<0.001
**Biochemistry**			
**BUN**	32.8 ± 6.4	28.9 ± 6.9	<0.001
**Creatinine**	1.3 ± 0.2	1.1 ± 0.2	<0.001
**Blood Pressure (mmHg)**			
**Systolic**	110.3 ± 22.1	99.0 ± 18.4	<0.001
**Diastolic**	70.7 ± 14.6	64.9 ± 11.5	<0.001
**MAP**	97.9 ± 12.3	89.6 ± 11.7	<0.001
**Dietary Intake**			
**Water (glass per day)**	5.0 ± 3.0	4.6 ± 3.3	0.103
**Salt (gram per day)**	2.8 ± 3.0	3.1 ± 3.7	0.186
**Ultrasound Measures (mm)**			
**RRL**	107.6 ± 8.6	103.8 ± 8.3	<0.001
**RRW**	43.7 ± 7.4	38.2 ± 6.0	<0.001
**LRL**	108.9 ± 7.9	104.1 ± 10.0	<0.001
**LRW **	47.9 ± 7.1	43.8 ± 6.8	<0.001

BMI, Body Mass Index; BSA, Body Surface Area; ICW, Intracellular Water; ECW, Extracellular Water; BUN, Blood Urea Nitrogen; MAP, Mean Arterial Pressure; RRL=Right Renal Length, LRL=Left Renal Length, RRW=Right Renal Width, LRW=Left Renal Width

**Notes:** Values are represented as the mean ± SD or N (%). BSA was calculated using the DuBois formula. Statistical analysis via independent - test or chi-square.

**Table 2:** Normal values of the kidney measurements classified Bbsed on age and gender category

Gender	Measures (mm)	30-40 (N= 175)	41-50(N=179)	51-60(N=83)	61-70(N=7)	P-value
Male	RRL	108.6±8.1 (83-130)	106.5±8.9 (86-130)	106.6±7.6 (90-132)	102.6±7.3 (95-112)	0.062
	RRE	43.9±7.5 (31-91)	42.7±6.6 (26-65)	43.1±8.4 (28-99)	43.3±6.9 (30-53)	0.671
	RR APD	46.1±6.6 (29-71)	45.6±5.9 (32-62)	45.5±5.7 (35-60)	47.1±4.8 (42-52)	0.815
	RRP	14.9±2.8 (9.7-26)	14.8±2.6 (9.3-22)	14.6±2.7 (9-23.7)	14.3±2.6 (10-8.3)	0.695
	LRL	109.0±7.7 (86-133)	109.1±7.6 (94-132)	107.6±7.2 (94-129)	102.0±9.7 (86-114)	0.062
	LRW	48.1±6.7 (30-64)	47.4±6.7 (30-66)	46.5±7.4 (10-63)	45.9±5.4 (38-55)	0.360
	LR APD	46.9±5.8 (33-64)	47.0±7.1 (18-67)	46.9±6.3 (28-64.5)	45.9±7.3 (31-54)	0.973
	LRP	15.7±3.0 (8.7-28)	15.4±2.8 (9-29)	15.4±2.7 (9-24.7)	15.0±3.1 (10-1.3)	0.706
		30-40 (N=235)	41-50(N=193)	51-60(N=56)	61-70(N=10)	P-value
Female	RRL	104.3±8.3 (84-126)	104.7±8.4 (85-130)	100.8±7.2 (85-117)	99.2±7.8 (85-108)	0.004
	RRW	37.8±5.8 (22-56)	38.5±6.2 (25-63)	37.7±5.8 (27-53)	39.6±7.5 (23-47)	0.570
	RR APD	42.7±6.2 (17-60)	42.9±6.2 (28-65)	43.8±5.5 (32-59)	43.6±6.1 (35-55)	0.712
	RRP	13.8±2.7 (9-24)	13.9±2.6 (6.3-21.3)	13.2±2.3 (8.3-18.5)	13.8±3.1 (10-19.5)	0.219
	LRL	104.3±0.4 (11-132)	104.9±10.8 (10-123)	102.0±0.1 (88-117)	96.6±6.7 (90-111)	**0.039**
	LRW	44.1±6.9 (13-58)	42.9±7.0 (11-59)	44.8±5.9 (28-57)	40.7±7.3 (27-50)	0.095
	LR APD	44.5±5.2 (33-59)	44.3±6.1 (7-62)	44.1±5.6 (30-58)	42.7±7.1 (30-56)	0.757
	LRP	14.5±2.7 (7.3-24.7)	14.7±2.7 (8.7-23.3)	14.1±2.5 (8.3-19.7)	14.5± .9 (9.7-8.3)	0.417

RRL=Right Renal Length, LRL=Left Renal Length, RRW=Right Renal Width, LRW=Left Renal Width, RRT=Right Renal Parenchymal Thickness, LRP=Left Renal Parenchymal Thickness, RR APD=Right Renal Anteroposterior Diameter, LR APD=Left Renal Anteroposterior Diameter;

**Notes:** Values are represented as mean ± SD (min-max).

**Table 3:** Normal values of the kidney measurements classified based on the height and gender category

Gender	Measures (mm)	140-150 (N=0)	151-160(N=13)	161-170(N=131)	171-180(N=253)	>180(N=47)	P-value
Male	RRL	N/A	102 ± 6.6 (90-110)	106 ± 8.1 (86-130)	107 ± 8.2 (83-130)	112 ± 7.9 (97-132)	**<0.001**
	RRW	N/A	41 ± 7.5 (30-53)	43 ± 5.6 (30-63)	43 ± 8.4 (26-99)	46 ± 6.4 (32-58)	0.073
	RR APD	N/A	44 ± 7.0 (29-56)	45 ± 6.0 (32-67)	46 ± 6.0 (33-71)	48 ± 6.3 (36-62)	0.065
	RRP	N/A	13.2 ± 2.0 (10-17.3)	14.7 ± 2.6 (9-26)	14.7 ± 2.6 (9.3-25.5)	15.7 ± 3.0 (10-23.7)	**0.004**
	LRL	N/A	102 ± 9.1 (86-116)	108 ± 7.1 (93-125)	109 ± 7.8 (86-133)	111 ± 6.7 (95-128)	**0.001**
	LRW	N/A	46 ± 5.5 (38-55)	47 ± 7.5 (10-61)	47 ± 6.4 (30-66)	50 ± 7.0 (30-64)	0.086
	LR APD	N/A	46 ± 4.2 (41-53)	46 ± 5.7 (31-65)	47 ± 6.9 (18-67)	49 ± 6.2 (35-64)	0.109
	LRP	N/A	13.7 ± 2.4 (9-17)	15.5 ± 2.9 (8.7-28)	15.4 ± 2.7 (9-29)	16.2 ± 2.9 (9.7-25.2)	**0.009**
		**140-150(N=31)**	**151-160(N=271)**	**161-170** **(N=183)**	**171-180(N=9)**	**>180(N=0)**	**P-value**
Female	RRL	100 ± 7.9 (85-120)	103 ± 7.7 (84-130)	106 ± 8.6 (86-128)	109 ± 5.6 (100-114)	N/A	**<0.001**
	RRW	36 ± 5.2 (23-49)	38 ± 6.2 (22-61)	38 ± 5.8 (25-63)	39 ± 6.5 (30-46)	N/A	0.083
	RR APD	40 ± 4.1 (32-50)	43 ± 5.9 (30-60)	43 ± 6.5 (17-65)	41 ± 6.9 (34-51)	N/A	0.067
	RRP	12.9±2.7 (8.3-19.7)	13.7 ± 2.5 (6.3-21.7)	13.9 ± 2.8 (7.3-24)	14.7 ± 2.7 (10.3-17.5)	N/A	0.226
	LRL	96±17.1 (10-113)	104 ± 7.5 (83-123)	106 ± 11.2 (11-132)	111 ± 6.1 (103-120)	N/A	**<0.001**
	LRW	43 ± 7.1 (27-56)	44 ± 7.0 (11-59)	44 ± 6.6 (16-57)	45 ± 3.1 (42-50)	N/A	0.951
	LR APD	42 ± 4.8 (30-54)	44 ± 5.5 (30-59)	45 ± 5.9 (7-62)	46 ± 3.1 (42-49)	N/A	0.158
	LRP	14.2±2.4 (9.3-20)	14.5 ± 2.5 (7.3-22)	14.6 ± 2.9 (8-24.7)	16.1 ± 2.3 (12.7-19.3)	N/A	0.513

RRL=Right Renal Length, LRL=Left Renal Length, RRW=Right Renal Width, LRW=Left Renal Width, RRT=Right Renal Parenchymal Thickness, LRP=Left Renal Parenchymal Thickness, RR APD=Right Renal Anteroposterior Diameter, LR APD=Left Renal Anteroposterior Diameter;

**Notes:** Values are represented as mean ± SD (min-max).

**Table 4:** Normal values of the kidney measurements classified based on the gender and weight category

Gender	Measures (mm)	<60 *(N=11)	60-70(N=85)	71-80(N=130)	81-90(N=145)	91-100(N=55)	>100(N=18)	P-value
Male	RRL	98 ± 8.0 (86-112)	104 ± 7.3 (83-117)	106 ± 7.1 (87-125)	109 ± 7.5 (92-130)	112 ± 8.4 (92-132)	115 ± 9.6 (93-128)	**<0.001**
	RRW	37 ± 6.4 (30-50)	40 ± 5.6 (26-53)	42 ± 8.8 (30-99)	45 ± 5.6 (34-65)	47 ± 6.3 (34-65)	48 ± 5.3 (38-58)	**<0.001**
	RR APD	39 ± 6.0 (29-48)	44 ± 4.9 (36-55)	45 ± 6.1 (33-71)	46 ± 5.8 (35-67)	49 ± 6.3 (39-64)	50 ± 6.0 (40-60)	**<0.001**
	RRP	13.6 ± 2.4 (9.3-18)	14.0 ± 2.3 (9.7-20)	14.2 ± 2.5 (9.7-20.3)	15.3 ± 2.7 (9-26)	15.2 ± 3.0 (10-25.5)	16.7 ± 3.0 (12-23.7)	**<0.001**
	LRL	99 ± 5.6 (93-112)	106 ± 6.7 (92-119)	107 ± 6.6 (86-124)	110 ± 7.0 (95-130)	113 ± 8.6 (86-133)	117 ± 5.9 (108-129)	**<0.001**
	LRW	43 ± 4.3 (38-52)	44 ± 7.9 (10-58)	46 ± 6.4 (30-63)	49 ± 5.7 (32-65)	51 ± 6.7 (35-66)	49 ± 4.7 (41-56)	**<0.001**
	LR APD	42 ± 5.7 (31-53)	45 ± 6.2 (28-65)	46 ± 6.3 (18-59)	48 ± 6.1 (33-67)	50 ± 6.3 (38-65)	50 ± 6.0 (40-56)	**<0.001**
	LRP	14.1 ± 2.5 (9-18.5)	14.9 ± 2.7 (8.7-24.7)	15.2 ± 3.0 (9-29)	15.7 ± 2.6 (9.7-28)	15.9 ± 3.1 (10.7-25.2)	17.0 ± 2.7 (12-20.3)	**0.005**
		**<60 (N=116)**	**60-70(N=151)**	**71-80(N=148)**	**81-90(N=58)**	**91-100(N=17)**	**>100(N=4** ** (**	**P-value**
Female	RRL	101 ± 6.5 (86-118)	103 ± 7.8 (84-125)	106 ± 7.9 (89-124)	110 ± 8.5 (91-128)	109 ± 11.3 (85-130)	115 ± 12.6 (103-128)	**<0.001**
	RRW	36 ± 5.0 (22-53)	37 ± 5.7 (23-56)	40 ± 5.2 (27-53)	42 ± 7.1 (25-63)	46 ± 6.3 (36-61)	39 ± 6.5 (34-48)	**<0.001**
	RR APD	42 ± 5.8 (30-57)	42 ± 5.8 (28-59)	44 ± 6.5 (17-59)	44 ± 5.2 (34-58)	46 ± 5.5 (40-60)	50 ± 10.5 (42-65)	**0.002**
	RRP	10.6 ± 0.4 (10.3-10.8)	13.0 ± 2.6 (8.3-16.8)	13.7 ± 2.5 (9.3-21.2)	13.3 ± 2.5 (6.3-21)	14.2 ± 2.7 (8.3-24)	15.0 ± 3.4 (11.7-19)	**0.018**
	LRL	100 ± 11.1 (10-119)	104 ± 10.8 (11-123)	106 ± 7.2 (87-121)	108 ± 7.3 (93-122)	110 ± 10.9 (90-132)	110 ± 7.4 (100-117)	**<0.001**
	LRW	43 ± 7.1 (13-57)	43 ± 7.0 (11-59)	45 ± 6.3 (30-58)	46 ± 4.8 ( 38-57)	46 ± 8.0 (30-54)	42 ± 6.6 (34-48)	**0.013**
	LR APD	43 ± 5.1 (30-58)	44 ± 6.0 (7-59)	45 ± 5.6 (31-59)	46 ± 4.7 (32-57)	46 ± 4.6 (38-54)	47 ± 10.3 (40-62)	**0.047**
	LRP	12.2 ± 4.0 (9.3-15)	13.8 ± 1.7 (11.7-17)	14.4 ± 2.5 (7.3-20)	14.4 ± 2.6 (9-23.3)	14.8 ± 2.9 (8.3-24.7)	15.3 ± 2.3 (13.3-17.3)	0.374

RRL=Right Renal Length, LRL=Left Renal Length, RRW=Right Renal Width, LRW=Left Renal Width, RRT=Right Renal Parenchymal Thickness, LRP=Left Renal Parenchymal Thickness, RR APD=Right Renal Anteroposterior Diameter, LR APD=Left Renal Anteroposterior Diameter; * kilogram

**Notes:** Values are represented as mean ± SD.

**Table 5:** Correlation of the potential risk factors with kidney measures

	RK length	RK width	RK AP	LK length	LK width	LK AP
**Age**	r=-0.040, P=0.219	r=0.095**, P=0.004	r=0.040, P=0.224	r=-0.024, P=0.467	r=0.021, P=0.528	r=0.018, P=0.585
**Height **	r=0.315**, P=0.000	r=0.339**, P=0.000	r=0.205**, P=0.000	r=0.305**, P=0.000	r=0.240**, P=0.000	r=0.220**, P=0.000
**Weight**	r=0.448**, P=0.000	r=0.450**, P=0.000	r=0.310**, P=0.000	r=0.415**, P=0.000	r=0.336**, P=0.000	r=0.302**, P=0.000
**BMI**	r=0.291**, P=0.000	r=0.275**, P=0.000	r=0.211**, P=0.000	r=0.258**, P=0.000	r=0.213**, P=0.000	r=0.191**, P=0.000
**BSA**	r=0.445**, P=0.000	r=0.457**, P=0.000	r=0.303**, P=0.000	r=0.418**, P=0.000	r=0.336**, P=0.000	r=0.304**, P=0.000
**Abdomen Circumference**	r=0.358**, P=0.000	r=0.369**, P=0.000	r=0.262**, P=0.000	r=0.330**, P=0.000	r=0.266**, P=0.000	r=0.241**, P=0.000
**Total Body Water**	r=0.407**, P=0.000	r=0.447**, P=0.000	r=0.288**, P=0.000	r=0.399**, P=0.000	r=0.343**, P=0.000	r=0.287**, P=0.000
**ICW**	r=0.406**, P=0.000	r=0.446**, P=0.000	r=0.287**, P=0.000	r=0.398**, P=0.000	r=0.344**, P=0.000	r=0.286**, P=0.000
**ECW**	r=0.408**, P=0.000	r=0.448**, P=0.000	r=0.289**, P=0.000	r=0.399**, P=0.000	r=0.338**, P=0.000	r=0.288**, P=0.000
**Mineral (kg)**	r=0.410**, P=0.000	r=0.431**, P=0.000	r=0.281**, P=0.000	r=0.394**, P=0.000	r=0.330**, P=0.000	r=0.282**, P=0.000
**Protein (kg)**	r=0.406**, P=0.000	r=0.446**, P=0.000	r=0.286**, P=0.000	r=0.399**, P=0.000	r=0.343**, P=0.000	r=0.285**, P=0.000
**BUN**	r=-0.033, P=0.330	r=0.058, P=0.083	r=0.049, P=0.150	r=0.003, P=0.928	r=0.067*, P=0.048	r=0.052, P=0.123
**Creatinine**	r=0.014, P=0.667	r=0.194**, P=0.000	r=0.112**, P=0.001	r=0.072*, P=0.032	r=0.106**, P=0.002	r=0.070*, P=0.037
**Systolic**	r=0.182**, P=0.000	r=0.155**, P=0.000	r=0.134**, P=0.000	r=0.165**, P=0.000	r=0.137**, P=0.000	r=0.097**, P=0.003
**Diastolic**	r=0.164**, P=0.000	r=0.151**, P=0.000	r=0.102**, P=0.002	r=0.139**, P=0.000	r=0.109**, P=0.001	r=0.076*, P=0.021
**MAP**	r=0.165**, P=0.000	r=0.184**, P=0.000	r=0.123**, P=0.000	r=0.186**, P=0.000	r=0.171**, P=0.000	r=0.081*, P=0.015
**Water Intake**	r=0.099**, P=.002	r=0.085**, P=.009	r=0.041, P=0.209	r=0.078*, P=0.017	r=0.134**, P=0.000	r=0.093**, P=0.005
**Salt Intake**	r=0.048, P=0.145	r=0.019, P=0.567	r=0.036, P=0.273	r=0.001, P=0.967	r=0.013, P=0.698	r=0.024, P=0.458

BMI=Body Mass Index; BSA=Body Surface Area; ICW=Intracellular Water; ECW= Extracellular Water; BUN=Blood Urea Nitrogen; MAP=Mean Arterial Pressure; RK, Right Kidney; LK, Left Kidney Notes: **Correlation is significant at the 0.01 level (2-tailed). *Correlation is significant at the 0.05 level (2-tailed).

**Table 6:** Predictors of the kidney length (mean length of the right and left kidneys)

Variable	Bivariate Analyses		Multivariate Analyses	
	β (SE)	P	β (SE)	P
**Age (year)**	-0.036 (0.034)	0.287		
**Height**	0.288 (0.025)	<0.001	-0.106 (0.057)	0.06
**Weight**	0.290 (0.017)	<0.001	0.189 (0.029)	<0.001
**Abdomen Circumference (cm)**	0.290 (0.022)	<0.001		
**Total Body Water (L)**	0.463 (0.029)	<0.001		
**Mineral (kg)**	5.094 (0.323)	<0.001	3.703 (0.989)	<0.001
**Protein (kg)**	1.689 (0.107)	<0.001		
**BUN**	-0.020 (0.038)	0.601		
**Creatinine**	1.801 (1.201)	0.134		
**Systolic BP**	0.074 (0.012)	<0.001		
**Diastolic BP**	0.101 (0.019)	<0.001		
**MAP**	0.125 (0.020)	<0.001		
**Water Intake (glass per day)**	0.255 (0.082)	0.002		
**Salt Intake (gram per day)**	0.056 (0.076)	0.460		

BUN, Blood Urea Nitrogen; MAP, Mean Arterial Pressure.

**Notes:** To adjust for multiple pairwise comparisons, P<.01 is considered statistically significant. Using forward stepwise regression, 3 factors (height, weight, and mineral) were found to be associated with Kidney Width. The R^2^ value for the final model was 0.27 (β=97.1).

## Discussion

This study aimed to evaluate the normal renal size in the healthy Iranian adult population, and also to explore the possible correlation of the influencing factors on kidney size. There are discrepant reports about normal kidney size in different populations (4, 7). Nonetheless, few similar studies have been conducted to provide evidence for the standard kidney size in healthy populations and most of the studies have not excluded the conditions affecting renal size, such as diabetes and hypertension (8). 

Although this variety is considered to be ethnic, we cannot only blame genetics. The environmental factors should also be considered as a significant factor (9). Therefore, it does not seem possible to determine an absolute number for normal kidney size. As mentioned earlier, measurement of kidney length is commonly used for diagnosis and treatment of kidney diseases and has a special place in the treatment, timely diagnosis, and preventing side effects (2). Our results showed a significant difference in kidney size between men and women so, the male renal size was significantly larger (table1).As there are conflicting reports on the correlation of kidney size with age (9-11) we also investigated this relationship. Our findings revealed aging and RRW had a  significant positive correlation (table 5). When considering sex, the female population showed a higher RRL (p=0.004) and LRL (P=0.039) between the age of 41-50 years, while male kidney size was not significantly different regarding to the study’s age groups (table 2). In a similar study by Jabbari et al., the analysis of different age groups, regardless of gender, showed a significant decrease in kidney length and parenchymal thickness after the age of fifty, according to their proportion of study population, which mostly included women, we have obtained the same results in our female population (9). In the study of Raza et al. in Pakistan, the relationship between the mean kidney length and age was found to be significant (11). Glodny et al. reported that kidney length increased significantly until the fifth decade of life in men (10). According to differences between age groups of our study, the resulting discrepancy may be explained.

Based on our study, height had a significant and positive correlation with all renal measurements (table 5, figures 2,3), Just as many other studies with a large population reported (10-12), Also, some small studies described no correlation (8,13). As for sex differences, within the male population, length, and parenchymal thickness in both kidneys and in the female group, both renal lengths had a significant positive relation with body height. Since weight and BMI had a significant positive correlation with kidney size, regarding gender, weight was also related to all renal measurements (except female LRP), as many other authors had mentioned (8-12,14). In a study by Muthusami et al., a moderate positive correlation was reported between kidney length with body weight and BSA while they estimated a poor positive correlation with height and BMI (7). 

According to our study, intracellular water, extracellular water, protein, and minerals also had a significant relationship with kidney measurement parameters. Furthermore, there was a positive and significant relationship between water consumption in the studied subjects and both kidneys’ lengths and width and left anteroposterior diameter. However, no significant relationship was observed between the amount of salt diet and any of the mentioned variables. 

To resolve the confounding effect of other variables, we performed the univariate and multivariate regression analysis. According to the studied variables, three variables including height, weight, and minerals remained significantly associated with kidney length (table 6). Su H-A et al. reported the association of the kidney length with weight and height (4). Also, Glondy et al. mentioned the same link for renal length and body height extracted from regression analysis (10). 

The value of the determination coefficient for the final model was 0.27 (β = 97.1), which indicates the prediction of 27% of changes in kidney thickness by these three variables. This study was conducted to assign reference ranges for normal kidney size in the Iranian adult population and to assess its possible influencing factors. The reference ranges presented in this study, as well as others, can be considered for implications in clinical guidelines or practice, however, in our opinion looking for an absolute normal kidney size definition is an unreasonable effort regarding its dynamic nature implying multiple factors capable to modify kidney size. We recommend researchers, clinicians, and radiologists to define a normal kidney size for every person separately and individually concerning factors including sex, age, underlying diseases, anthropometric characteristics, and diet. Future studies are suggested to explore the role of such modifying factors with regard to race in larger populations with a long-term follow-up design. 
